# Implementing very‐low‐dose allergen exposure after suspected IgE‐mediated reactions in infancy prior to allergist consultation

**DOI:** 10.1111/pai.70432

**Published:** 2026-07-10

**Authors:** Anna Voia, Roxane Labrosse, Kathryn Samaan, François Graham, Louis Paradis, Florence Gingras‐Lessard, Anne Des Roches, Philippe Bégin

**Affiliations:** ^1^ Allergy Section, Department of Pediatrics CHU Sainte‐Justine Montréal Canada; ^2^ Allergy Section, Department of Medicine Chum Montréal Canada

**Keywords:** digital consultation, early allergen exposure, food allergy, IgE‐mediated reactions, primary care, quality improvement

To the Editor,

Early allergen exposure in infancy is increasingly recognized as a critical opportunity to influence the trajectory of food allergy and possibly promote immune tolerance when introduced during a key developmental window.^1^, ^2^, ^3^, ^4^, ^5^ However, after a suspected immunoglobulin E–mediated reaction, infants are frequently advised to strictly avoid the culprit food while awaiting specialist assessment, despite often prolonged delays in access to allergy care.^6^ This creates an important system‐level gap between first presentation and specialist management, during a period in which missed opportunities for immune modulation may be clinically meaningful.

Family physicians and nurse practitioners are uniquely positioned to bridge this gap, as they are the first point of contact for most infants presenting with a suspected food‐allergic reaction. Yet, recommending re‐exposure after an immunoglobulin E–mediated reaction remains a substantial departure from traditional avoidance‐based paradigms and may generate discomfort for clinicians and families alike. Beyond clinical evidence, implementation of such an approach in routine primary care depends on provider confidence, clarity of recommendations, and family adherence.

We therefore conducted a quality improvement study to evaluate the real‐world implementation of a primary care–led interim management strategy based on very‐low‐dose allergen exposure while awaiting specialist allergy care. The study was conducted within a provincial asynchronous specialist support pathway in Quebec^7^, ^8^ through which primary care providers could seek allergist advice for infants presenting with suspected food‐allergic reactions. As part of the consulting allergist's clinical practice, selected infants were advised to maintain a fixed daily very‐low‐dose exposure, generally ranging from 4 to 10 mg of allergen protein, without dose escalation until in‐person allergy assessment.

Using a cross‐sectional survey design aligned with quality improvement reporting standards,^9^ we assessed provider uptake of recommendations, family adherence, short‐term outcomes, and barriers and facilitators to implementation in routine care. Quantitative process measures included whether the recommendation was communicated to families, implemented in practice, and maintained over time while awaiting specialist assessment. Outcome measures included the occurrence and severity of reactions after re‐exposure, continuation of daily ingestion, and subsequent specialist follow‐up. Qualitative responses were analyzed thematically to identify barriers and facilitators to implementation, with particular attention to provider comfort, family adherence, and contextual influences on uptake. This mixed‐methods quality improvement approach was chosen to capture both measurable implementation outcomes and the underlying mechanisms influencing real‐world adoption in routine primary care settings.

This quality improvement project was reviewed and approved by the institutional quality improvement committee and the provincial public health authority overseeing the digital consultation platform. Participation in the physician survey was voluntary, and completion of the survey implied informed consent.

Among 73 primary care providers contacted, 30 responded to the survey (41%), describing 34 infants. Most respondents were family physicians, with a minority of nurse practitioners, and most index reactions were mild and limited to a single organ system (Table 1). Following allergist guidance, 28 of 30 responding clinicians reported recommending very‐low‐dose allergen exposure to families. Importantly, 16 of these 28 clinicians subsequently reported making similar recommendations to other infants outside the original case context, suggesting early diffusion of this practice beyond the initial specialist‐supported interaction.

This finding is particularly relevant from an implementation perspective, as it suggests that the intervention was not perceived as a one‐off specialist‐directed exception but was sufficiently understood and accepted to be transferred to subsequent clinical situations. Beyond immediate uptake, the recommendation appears to have generated early clinician confidence and practice diffusion within primary care, representing a strong signal of real‐world feasibility and potential scalability of this care pathway.

**TABLE 1:** Participant characteristics.

Characteristic	*n*/*N* (%) or mean ± SD
Primary care providers (*n* = 30)	
Family physicians	26/30 (87)
Nurse practitioners	4/30 (13)
Infants described (*n* = 34)	
Age at index reaction, years	0.7 ± 2.9
Suspected culprit food	
Peanut	10/34 (29)
Egg	10/34 (29)
Milk	5/34 (15)
Other	10/34 (29)
Reaction severity	
Mild, single‐system	27/34 (79)
Moderate or severe	5/34 (15)
Not specified	2/34 (6)
Families receiving early exposure recommendation	31/34 (91)
Families following recommendations	24/31 (77)
Any reaction reported after exposure	4/24 (17)
Mild, single‐system reaction	4/4 (100)
Moderate or severe reaction	0
Epinephrine use	0
Families not following recommendation	5/31 (16)
From fear of reaction	3/5 (60)
Already had appointment with specialist	2/5 (40)
Unknown if family followed recommendation	2/31 (6)
Infants eventually seen by allergist	11/34 (32)
Subsequent allergist recommendations in infants who introduced (*n* = 7)	
Negative skin tests ‐ proceed with full introduction	2/7 (28)
Continued exposure / desensitization	4/7 (57)
Unknown	1/7 (14)
Subsequent allergist recommendations in infants who did not introduced (*n* = 4)	
Strict avoidance	1/4 (25)
Started on low‐dose immunotherapy	1/4 (25)
Referred to structured oral immunotherapy program	1/4 (25)
Unknown	1/4 (25)

Among the 31 families to whom the recommendation was communicated, at least 24 were reported to have implemented it, and most were able to maintain this approach during the waiting period before specialist evaluation. Short‐term outcomes remained reassuring, with most infants experiencing no reaction after re‐exposure. Four families reported a reaction after re‐exposure (three episodes of perioral urticaria and one eczema flare), all of which were mild and limited to a single organ system, with no use of epinephrine. The four infants who reacted had all presented initially with isolated perioral urticaria. Importantly, we are aware of at least one systemic cutaneous reaction among non‐responding cases managed through this pathway, underscoring the possibility of incomplete outcome ascertainment in this survey‐based study. In that case, the reaction occurring at such a low dose served as a clinically meaningful signal to prioritize earlier specialist allergy assessment. This case also illustrates that the pathway implicitly relied on continued specialist support, whereby primary care providers could implement the recommendation with the understanding that rapid escalation and coordination of regional allergy follow‐up would be actively supported if a reaction occurred.

The principal finding of this study is the real‐world feasibility of this interim primary care–led strategy, while also highlighting that the main challenge lies in implementation rather than access to specialist advice alone. Thematic analysis identified barriers at multiple levels, including parental fear of reactions, difficulties integrating daily dosing into family routines, uncertainty regarding dose calculation, and limited familiarity with food allergy management in primary care (Table 2). These findings emphasize that successful uptake depends not only on the recommendation itself but on its practical implementation by clinicians and families in real‐world care settings.

**TABLE 2:** Primary care provider's perspectives on early allergen exposure.

Theme	Subtheme	Illustrative quote
Digital allergist support as an implementation facilitator	Clear, actionable communication	“The digital advice gave me enough confidence to proceed with the exposure. The explanations were excellent, precise, and reassuring.” (ID#22)
	Specialist endorsement increasing parental trust	“Parents are often reassured knowing that we consulted an allergist before proceeding.” (ID#14)
	Overall satisfaction with the approach	“We followed the progressive exposure advice, and the redness gradually decreased.” (ID#1)
Need for parental guidance and practical support	Parental fear of allergic reactions	“Parents are often reluctant to reintroduce the potential allergen, even though it is actually beneficial for the child to resume it to induce tolerance!” (ID#13)
		“Parents fear an allergic reaction. However, with explanations, they clearly understand the need to continue exposure.” (ID#6)
	Challenges integrating exposure into daily routines	“There is difficulty including the allergen into the daily schedule: timing during the day, daycare departure, naps, and nighttime make it challenging to monitor allergic reactions in this context.” (ID#29)
	Difficulty administering precise doses	“It is difficult calculating the amount to give depending on the type of food.” (ID#17)
	Value of concrete tools and resources	“A clear introduction recipe is very helpful.” (ID#6)
	Desire for multimedia educational resources	“Online videos addressing these questions—practical, step‐by‐step guidance on how to manage allergen introduction.” (ID#29)
Training and knowledge gaps in primary care	Limited familiarity with food allergy management	“For most primary care providers, prescription of relevant blood tests and their interpretation is not routine.” (ID#11)
	Lack of confidence in the absence of formal diagnosis	“[There is a barrier] if there is diagnostic uncertainty.” (ID#19)
	Desire for additional training	“I would be very happy to participate in a conference on the topic with my colleagues in family medicine.” (ID#27)
Perceived inequities in access to allergy care influencing implementation	Moral conflict contributing to non‐implementation despite specialist digital support.	“No, I did not offer it to my patient. My personal experience: My 12‐year‐old daughter has multiple nut allergies. She previously had anaphylaxis to cashew, and recent testing confirms IgE levels of 100 for pistachio and cashew. The allergist suggested desensitization to almond, for which IgE was only 3. I have had to travel [long distance] regularly to increase doses. You can imagine my surprise when I read your response suggesting desensitizing a 6‐month‐old infant who had anaphylaxis in my clinic, when I cannot even progress doses or be told that my own child is not allergic [to almonds].” (ID#4)
	Perceived inconsistency between digital and in‐person specialist recommendations	“While the allergist from the digital support tool was very clear, the allergist who later saw the patient was more reluctant.” *(ID#28)*
	Concern that some patients may never access specialist follow‐up	“Barrier: Never getting a consultation.” (ID#27)

Importantly, this project was not designed to evaluate the efficacy or safety of very‐low‐dose allergen exposure itself. Rather, it sought to determine whether such recommendations could be operationalized within a primary care pathway supported by specialist consultation. Consequently, the absence of severe reactions in this cohort should not be interpreted as a definitive safety assessment. While the use of very‐low‐dose allergen exposure was informed by a broader body of evidence supporting low reactivity rates at very small allergen doses, rare severe reactions remain possible and would be expected to occur occasionally when such approaches are implemented at a larger population scale.

One of the most salient implementation barriers was the perceived heterogeneity of messages among allergists. Several respondents explicitly described discordance between the recommendations received through the specialist support pathway and those later provided during in‐person allergy consultation. This perceived inconsistency undermined clinician confidence and, in some cases, created uncertainty sufficient to limit implementation. In an implementation study, such inconsistency in specialist messaging may be as important as family adherence itself, as it directly influences the willingness of family physicians to adopt and sustain the practice.

This point is particularly important from a systems perspective. A care pathway that depends on primary care uptake cannot function optimally if specialist messaging varies substantially across settings. Alignment between asynchronous specialist advice and in‐person specialist care is therefore likely to be a key determinant of successful implementation.

The prolonged delays to in‐person allergy evaluation, illustrated in Figure 1, reinforce the clinical relevance of this issue. Although expected, the visual representation of these wait times confirms that many infants remain without specialist assessment during a period in which early intervention may be most relevant.^6^ In this context, the ability of primary care providers to initiate and families to maintain allergen exposure becomes particularly meaningful.

**FIGURE 1:**
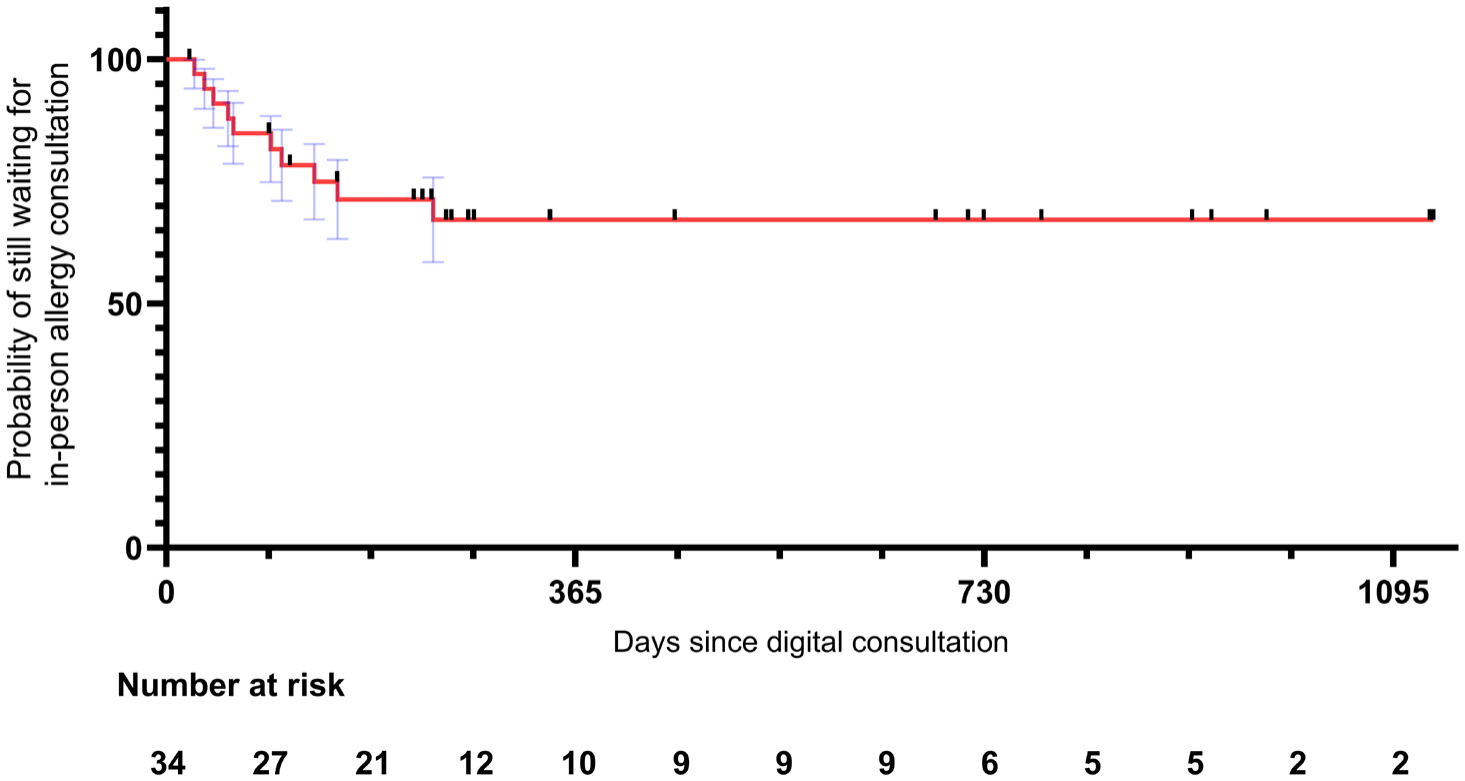
Time to in‐person allergist evaluation following digital consultation. Kaplan–Meier curve showing time from digital allergist consultation to first in‐person allergist assessment. Patients without an in‐person evaluation by the time of survey completion were censored. The wide distribution of event times and persistent censoring suggests substantial heterogeneity in access to in‐person allergy care across the sample, reinforcing the role of digital consultation as interim support during prolonged or variable wait times.

Taken together, these findings suggest that family physicians can play an active role in bridging the waiting period before specialist allergy assessment. However, successful implementation requires more than access to specialist advice. Alignment of messages across allergists and care settings, practical tools for dosing, educational support for families, and clinician training all appear essential to optimize uptake and reduce variability in practice.

These findings support the need for a more formalized and standardized care pathway across primary and specialist care settings to improve consistency of recommendations and clinician confidence.^10^

As a survey‐based quality improvement study, these findings should be interpreted within the limits of self‐reported clinician data and indirect reporting of patient outcomes; however, the convergence of quantitative uptake measures and qualitative implementation themes provides a coherent picture of real‐world feasibility and barriers in routine care.

This quality improvement study therefore supports the real‐world feasibility of a primary care–led interim allergen exposure strategy in infancy while also underscoring that implementation barriers, particularly heterogeneity in specialist messaging, remain critical targets for improvement.

## AUTHOR CONTRIBUTIONS

**Anna Voia:** Writing – review and editing; writing – original draft; investigation; formal analysis; data curation; methodology. **Philippe Bégin:** Conceptualization; funding acquisition; writing – original draft; methodology; investigation; formal analysis; project administration; data curation; supervision; writing – review and editing. **Anne Des Roches:** Conceptualization; writing – review and editing. **François Graham:** Conceptualization; writing – review and editing. **Louis Paradis:** Conceptualization; writing – review and editing. **Florence Gingras‐Lessard:** Conceptualization; writing – review and editing. **Kathryn Samaan:** Conceptualization; writing – review and editing. **Roxane Labrosse:** Conceptualization; writing – review and editing.

## FUNDING INFORMATION

P. Bégin is supported by the Fonds de Recherche du Québec (351936).

## CONFLICT OF INTEREST STATEMENT

P. Bégin reports professional relationships with Novartis, ALK, Viatris, DBV technologies and GSK unrelated to the content of this manuscript. P Bégin also serves as an Associate Editor for *Allergy, Asthma & Clinical Immunology*. Dr. Roxane Labrosse has received honoraria from AstraZeneca and Grifols for educational activities unrelated to the current manuscript. These activities did not influence the study design, data collection, analysis, interpretation, or reporting of the results. Other authors declare no financial or non‐financial conflicts of interest directly related to the work presented in this manuscript.

## ETHICS STATEMENT

This project was conducted as a quality improvement initiative and did not meet the criteria for research requiring formal research ethics board review under applicable institutional and provincial regulations. The project was reviewed and approved by the institutional Quality Improvement Committee and by the provincial Director of Public Health responsible for oversight of the digital consultation platform.

## CONSENT

Participation in the physician survey was voluntary, and completion of the survey implied informed consent.

## Data Availability

The datasets used and/or analyzed during the current study are available from the corresponding author on reasonable request.
